# (3*E*,5*E*)-3,5-Dibenzyl­idene-1-[3-(piperidin-1-yl)propano­yl]piperidin-4-one

**DOI:** 10.1107/S1600536811015984

**Published:** 2011-05-07

**Authors:** Yalda Kia, Hasnah Osman, Vikneswaran Murugaiyah, Madhukar Hemamalini, Hoong-Kun Fun

**Affiliations:** a School of Chemical Sciences, Universiti Sains Malaysia, 11800 USM, Penang, Malaysia; b School of Pharmaceutical Sciences, Universiti Sains Malaysia, 11800 USM, Penang, Malaysia; c X-ray Crystallography Unit, School of Physics, Universiti Sains Malaysia, 11800 USM, Penang, Malaysia

## Abstract

The asymmetric unit of the title compound, C_27_H_30_N_2_O_2_, comprises two independent mol­ecules. The dihedral angles between the phenyl rings in the two mol­ecules are 55.59 (8) and 55.39 (8)°. The piperidine rings adopt chair conformations. The crystal structure is stabilized by weak inter­molecular C—H⋯O and C—H⋯N hydrogen bonds. The crystal studied was a non-merohedral twin with a domian ratio of 0.75 (2):0.25 (2).

## Related literature

For details and applications of α, β-unsaturated ketones, see: Lee *et al.* (1971 ▶, 1977 ▶); Maria *et al.* (2000 ▶); Murakami *et al.* (2002 ▶); Kawase *et al.* (2002 ▶); Hitosugi *et al.* (2003 ▶). For the synthetic procedure of 1-acryloyl-3,5-dibenzyl­idene piperidin-4-one, see: Dimmock *et al.* (2000 ▶). For ring conformations, see: Cremer & Pople (1975 ▶). For bond-length data, see: Allen *et al.* (1987 ▶). For the stability of the temperature controller used in the data collection, see: Cosier & Glazer (1986 ▶).
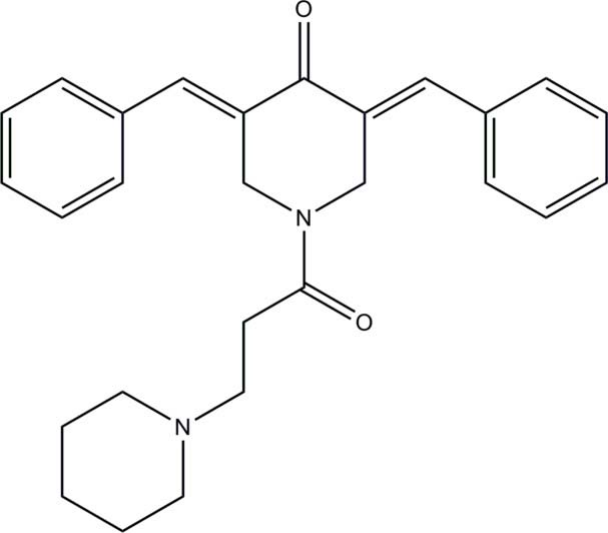


## Experimental

### 

#### Crystal data

C_27_H_30_N_2_O_2_
*M* *_r_* = 414.53
Triclinic, 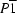

*a* = 9.7757 (6) Å
*b* = 10.9562 (6) Å
*c* = 20.9400 (15) Å
α = 93.065 (1)°
β = 96.594 (1)°
γ = 90.115 (1)°
*V* = 2224.7 (2) Å^3^
*Z* = 4
Mo *K*α radiation
μ = 0.08 mm^−1^
*T* = 100 K
0.62 × 0.15 × 0.07 mm

#### Data collection

Bruker APEXII DUO CCD area-detector diffractometer
Absorption correction: multi-scan (*SADABS*; Bruker, 2009 ▶) *T*_min_ = 0.953, *T*_max_ = 0.994
11714 measured reflections
11714 independent reflections
8608 reflections with *I* > 2σ(*I*)

#### Refinement

*R*[*F* ^2^ > 2σ(*F* ^2^)] = 0.046
*wR*(*F* ^2^) = 0.122
*S* = 1.04
11714 reflections
561 parameters
H-atom parameters constrained
Δρ_max_ = 0.35 e Å^−3^
Δρ_min_ = −0.23 e Å^−3^

### 

Data collection: *APEX2* (Bruker, 2009 ▶); cell refinement: *SAINT* (Bruker, 2009 ▶); data reduction: *SAINT*; program(s) used to solve structure: *SHELXTL* (Sheldrick, 2008 ▶); program(s) used to refine structure: *SHELXTL*; molecular graphics: *SHELXTL*; software used to prepare material for publication: *SHELXTL* and *PLATON* (Spek, 2009 ▶).

## Supplementary Material

Crystal structure: contains datablocks global, I. DOI: 10.1107/S1600536811015984/lh5234sup1.cif

Structure factors: contains datablocks I. DOI: 10.1107/S1600536811015984/lh5234Isup2.hkl

Supplementary material file. DOI: 10.1107/S1600536811015984/lh5234Isup3.cml

Additional supplementary materials:  crystallographic information; 3D view; checkCIF report

## Figures and Tables

**Table 1:** Hydrogen-bond geometry (Å, °)

*D*—H⋯*A*	*D*—H	H⋯*A*	*D*⋯*A*	*D*—H⋯*A*
C7*A*—H7*AB*⋯O1*B*^i^	0.97	2.59	3.3737 (19)	138
C19*B*—H19*B*⋯O2*A*^ii^	0.93	2.44	3.233 (2)	143
C24*A*—H24*A*⋯O2*B*^iii^	0.93	2.40	3.190 (2)	143
C24*B*—H24*B*⋯N2*A*	0.93	2.61	3.411 (2)	145

Symmetry codes: (i) 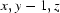; (ii) 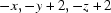; (iii) 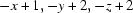.

**Fig. 1.**
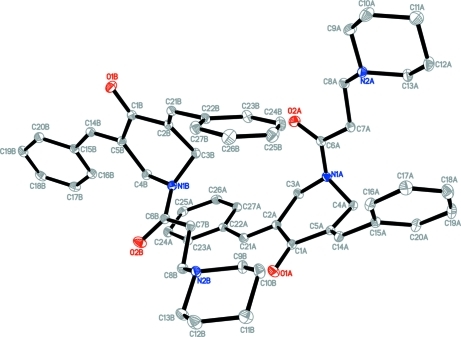
The asymmetric unit of the title compound, showing 30% probability displacement ellipsoids and the atom-numbering scheme. Hydrogen atoms omitted for clarity.

**Fig. 2.**
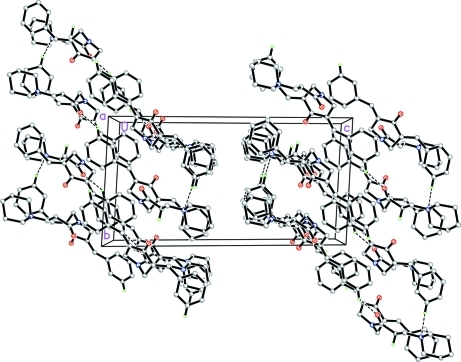
The crystal packing of the title compound (I) with hydrogen bonds shown as dashed lines. H atoms not involved in the intermolecular interactions have been omitted for clarity.

## supplementary crystallographic information

### Comment

α, β- Unsaturated ketones from Claisen-Schmidt condensation reactions between aldehydes and ketones display a wide variety of biological activities such as antimicrobial, antitumor and plant growth regulatory properties (Lee *et al.*, 1971, 1977; Maria *et al.*, 2000; Murakami *et al.*, 2002). The structure-activity relationship study of these compounds has shown that these activities are due to the presence of carbonyl group in these structures (Kawase *et al.*, 2002; Hitosugi *et al.*, 2003).  α, β- Unsaturated ketones can be considered as a Michael acceptor which is an active moiety showing enzyme inhibitory activity. Due to these reasons, the crystal structure determination of the title compound was carried out and the results are presented in this paper.

The asymmetric unit of the title compound, consists of two crystallographically independent (3*E*,5*E*)-3,5-Dibenzylidene-1- (3-(piperidin-1-yl)propanoyl)piperidin-4-one molecules, (A & B), as shown in Fig. 1. The bond lengths and angles of molecules A and B agree with each other and are within normal ranges for bond lengths (Allen *et al.*, 1987). The dihedral angles between terminal phenyl rings (C15A–C20A)/(C22A–C27A), and (C15B–C20B)/(C22B–C27B) are 55.59 (8) and 55.39 (8)°   respectively.  The piperidine rings adopts a chair conformation [(N1A/C1A–C5A); Q = 0.5186 (15) Å, θ = 129.31 (17)°, φ = 152.3 (2)°; (N2A/C9A–C13A); Q = 0.5756 (18) Å, θ = 2.71 (18)°, φ = 12 (4)°; and (N1B/C1B–C5B);  Q = 0.5207 (15) Å, θ = 128.88 (17)°, φ = 208.1 (2)°; (N2B/C9B–C13B); Q = 0.5764 (18) Å, θ = 1.07 (17)°, φ = 22 (8)°; Cremer & Pople, 1975].

In the crystal structure (Fig. 2), the molecules are linked through intermolecular C7A—H7AB···O1B^i^, C19B—H19B···O2A^ii^, C24A—H24A···O2B^iii^ and  C24B—H24B···N2A (see table 1 for symmetry codes Table 1) hydrogen bonds.

### Experimental

1-Acryloyl-3,5-dibenzylidenepiperidin-4-one (Dimmock *et al.*, 2000) were synthesized as reported in the literature. The title compound (I) was prepared by refluxing 1-acryloyl-3,5-dibenzylidenepiperidin-4-one (0.6 mmol) with piperidine (0.6 mmol) in ethanol. After completion of the reaction (through TLC monitoring), the mixture was poured into ice. The precipitated solid was filtered and washed with water. The pure solid was then recrystallised from ethanol to afford the title compound as yellow crystals.

### Refinement

All hydrogen atoms were positioned geometrically [C–H = 0.93 or 0.97 Å] and were refined using a riding model, with *U*_iso_(H) = 1.2 *U*_eq_(C). The crystal is a twin with twin law  1 0 0 0 -1 0 -0.5 0 -1 and BASF = 0.23. Six outliners 1 -2 7, 1 -3 8, 2 -3 3, -1 -4 2, -5 -6 2 and 3 -3 3 were omitted.

### Crystal data

**Table:**

C_27_H_30_N_2_O_2_	*Z* = 4
*M**_r_* = 414.53	*F*(000) = 888
Triclinic, *P*1	*D*_x_ = 1.238 Mg m^−^^3^
Hall symbol: -P 1	Mo *K*α radiation, λ = 0.71073 Å
*a* = 9.7757 (6) Å	Cell parameters from 8978 reflections
*b* = 10.9562 (6) Å	θ = 3.4–31.1°
*c* = 20.9400 (15) Å	µ = 0.08 mm^−^^1^
α = 93.065 (1)°	*T* = 100 K
β = 96.594 (1)°	PLATE, yellow
γ = 90.115 (1)°	0.62 × 0.15 × 0.07 mm
*V* = 2224.7 (2) Å^3^	

### Data collection

**Table:**

Bruker APEXII DUO CCD area-detector diffractometer	11714 independent reflections
Radiation source: fine-focus sealed tube	8608 reflections with *I* > 2σ(*I*)
graphite	*R*_int_ = 0.000
φ and ω scans	θ_max_ = 29.0°, θ_min_ = 2.1°
Absorption correction: multi-scan (*SADABS*; Bruker, 2009)	*h* = −13→13
*T*_min_ = 0.953, *T*_max_ = 0.994	*k* = −14→14
11714 measured reflections	*l* = −28→28

### Refinement

**Table:**

Refinement on *F*^2^	Primary atom site location: structure-invariant direct methods
Least-squares matrix: full	Secondary atom site location: difference Fourier map
*R*[*F*^2^ > 2σ(*F*^2^)] = 0.046	Hydrogen site location: inferred from neighbouring sites
*wR*(*F*^2^) = 0.122	H-atom parameters constrained
*S* = 1.04	*w* = 1/[σ^2^(*F*_o_^2^) + (0.0538*P*)^2^ + 0.3476*P*] where *P* = (*F*_o_^2^ + 2*F*_c_^2^)/3
11714 reflections	(Δ/σ)_max_ = 0.001
561 parameters	Δρ_max_ = 0.35 e Å^−^^3^
0 restraints	Δρ_min_ = −0.23 e Å^−^^3^

### Special details

**Table:**

Experimental. The crystal was placed in the cold stream of an Oxford Cryosystems Cobra open-flow nitrogen cryostat (Cosier & Glazer, 1986) operating at 100.0 (1) K.
Geometry. All s.u.'s (except the s.u. in the dihedral angle between two l.s. planes) are estimated using the full covariance matrix. The cell s.u.'s are taken into account individually in the estimation of s.u.'s in distances, angles and torsion angles; correlations between s.u.'s in cell parameters are only used when they are defined by crystal symmetry. An approximate (isotropic) treatment of cell s.u.'s is used for estimating s.u.'s involving l.s. planes.
Refinement. Refinement of F^2^ against ALL reflections. The weighted R-factor wR and goodness of fit S are based on F^2^, conventional R-factors R are based on F, with F set to zero for negative F^2^. The threshold expression of F^2^ > 2σ(F^2^) is used only for calculating R-factors(gt) etc. and is not relevant to the choice of reflections for refinement. R-factors based on F^2^ are statistically about twice as large as those based on F, and R- factors based on ALL data will be even larger.

### Fractional atomic coordinates and isotropic or equivalent isotropic displacement parameters (Å^2^)

**Table:**

	*x*	*y*	*z*	*U*_iso_*/*U*_eq_	
O1A	0.52837 (11)	0.52933 (11)	0.81181 (5)	0.0256 (2)	
O2A	−0.00087 (11)	0.44336 (11)	0.85115 (5)	0.0279 (3)	
N1A	0.20082 (12)	0.34156 (12)	0.85739 (6)	0.0182 (2)	
N2A	−0.15237 (13)	0.28130 (12)	0.67290 (6)	0.0192 (3)	
C1A	0.43904 (15)	0.46644 (14)	0.83063 (7)	0.0190 (3)	
C2A	0.37632 (15)	0.50083 (14)	0.89125 (7)	0.0185 (3)	
C3A	0.26373 (15)	0.42108 (14)	0.91089 (7)	0.0205 (3)	
H3AA	0.3019	0.3713	0.9454	0.025*	
H3AB	0.1934	0.4726	0.9271	0.025*	
C4A	0.30179 (15)	0.26758 (14)	0.82639 (7)	0.0188 (3)	
H4AA	0.2554	0.2070	0.7959	0.023*	
H4AB	0.3612	0.2256	0.8584	0.023*	
C5A	0.38504 (14)	0.35374 (14)	0.79221 (7)	0.0186 (3)	
C6A	0.07262 (15)	0.36533 (14)	0.82849 (7)	0.0195 (3)	
C7A	0.02468 (15)	0.28936 (14)	0.76711 (7)	0.0195 (3)	
H7AA	0.0831	0.3074	0.7343	0.023*	
H7AB	0.0346	0.2034	0.7754	0.023*	
C8A	−0.12417 (15)	0.31395 (15)	0.74207 (7)	0.0205 (3)	
H8AA	−0.1845	0.2669	0.7653	0.025*	
H8AB	−0.1436	0.3999	0.7499	0.025*	
C9A	−0.28999 (17)	0.32203 (17)	0.64931 (8)	0.0290 (4)	
H9AA	−0.2978	0.4088	0.6600	0.035*	
H9AB	−0.3576	0.2793	0.6705	0.035*	
C10A	−0.3201 (2)	0.29829 (18)	0.57705 (8)	0.0348 (4)	
H10A	−0.2564	0.3452	0.5556	0.042*	
H10B	−0.4127	0.3246	0.5629	0.042*	
C11A	−0.30604 (19)	0.16306 (18)	0.55875 (9)	0.0341 (4)	
H11A	−0.3777	0.1169	0.5754	0.041*	
H11B	−0.3165	0.1502	0.5123	0.041*	
C12A	−0.16569 (18)	0.11893 (16)	0.58650 (8)	0.0281 (4)	
H12A	−0.0948	0.1569	0.5652	0.034*	
H12B	−0.1604	0.0312	0.5785	0.034*	
C13A	−0.14007 (17)	0.14993 (14)	0.65860 (7)	0.0235 (3)	
H13A	−0.2062	0.1063	0.6803	0.028*	
H13B	−0.0486	0.1236	0.6748	0.028*	
C14A	0.39920 (16)	0.34674 (14)	0.72900 (7)	0.0208 (3)	
H14A	0.4486	0.4100	0.7143	0.025*	
C15A	0.34632 (15)	0.25153 (15)	0.68040 (7)	0.0204 (3)	
C16A	0.28336 (16)	0.28818 (15)	0.62121 (7)	0.0229 (3)	
H16A	0.2780	0.3710	0.6136	0.028*	
C17A	0.22888 (18)	0.20310 (17)	0.57379 (8)	0.0276 (4)	
H17A	0.1840	0.2287	0.5353	0.033*	
C18A	0.24164 (19)	0.07926 (17)	0.58404 (8)	0.0305 (4)	
H18A	0.2056	0.0219	0.5522	0.037*	
C19A	0.30775 (18)	0.04082 (16)	0.64148 (8)	0.0299 (4)	
H19A	0.3181	−0.0422	0.6476	0.036*	
C20A	0.35868 (17)	0.12620 (15)	0.68999 (8)	0.0248 (3)	
H20A	0.4010	0.1001	0.7289	0.030*	
C21A	0.42705 (15)	0.60311 (14)	0.92446 (7)	0.0197 (3)	
H21A	0.4946	0.6431	0.9055	0.024*	
C22A	0.39430 (15)	0.66211 (14)	0.98519 (7)	0.0193 (3)	
C23A	0.44431 (16)	0.78206 (14)	1.00010 (8)	0.0234 (3)	
H23A	0.4955	0.8193	0.9716	0.028*	
C24A	0.41863 (17)	0.84563 (16)	1.05642 (8)	0.0263 (3)	
H24A	0.4518	0.9251	1.0653	0.032*	
C25A	0.34369 (16)	0.79093 (16)	1.09947 (8)	0.0270 (4)	
H25A	0.3255	0.8340	1.1370	0.032*	
C26A	0.29564 (16)	0.67184 (16)	1.08672 (7)	0.0256 (3)	
H26A	0.2462	0.6349	1.1159	0.031*	
C27A	0.32149 (15)	0.60788 (15)	1.03025 (7)	0.0222 (3)	
H27A	0.2899	0.5278	1.0223	0.027*	
O1B	−0.11562 (11)	1.02694 (10)	0.80768 (5)	0.0253 (2)	
O2B	0.42975 (11)	0.94230 (11)	0.85129 (6)	0.0294 (3)	
N1B	0.23096 (12)	0.83864 (12)	0.85633 (6)	0.0186 (3)	
N2B	0.50078 (13)	0.78183 (12)	0.67358 (6)	0.0184 (3)	
C1B	−0.01801 (15)	0.96405 (14)	0.82724 (7)	0.0189 (3)	
C2B	0.01804 (14)	0.85146 (13)	0.78926 (7)	0.0183 (3)	
C3B	0.11570 (15)	0.76496 (14)	0.82443 (7)	0.0190 (3)	
H3BA	0.0700	0.7233	0.8559	0.023*	
H3BB	0.1480	0.7042	0.7944	0.023*	
C4B	0.19298 (15)	0.91820 (14)	0.90957 (7)	0.0198 (3)	
H4BA	0.2711	0.9697	0.9264	0.024*	
H4BB	0.1702	0.8685	0.9439	0.024*	
C5B	0.07126 (15)	0.99822 (14)	0.88884 (7)	0.0181 (3)	
C6B	0.34547 (15)	0.86392 (14)	0.82786 (7)	0.0199 (3)	
C7B	0.36501 (15)	0.78944 (14)	0.76644 (7)	0.0197 (3)	
H7BA	0.2912	0.8076	0.7333	0.024*	
H7BB	0.3592	0.7032	0.7742	0.024*	
C8B	0.50266 (15)	0.81580 (15)	0.74247 (7)	0.0207 (3)	
H8BA	0.5243	0.9022	0.7499	0.025*	
H8BB	0.5743	0.7705	0.7667	0.025*	
C9B	0.48466 (17)	0.64938 (14)	0.66087 (7)	0.0223 (3)	
H9BA	0.3989	0.6227	0.6750	0.027*	
H9BB	0.5595	0.6080	0.6852	0.027*	
C10B	0.48454 (17)	0.61487 (16)	0.58927 (7)	0.0265 (3)	
H10C	0.4762	0.5268	0.5822	0.032*	
H10D	0.4058	0.6515	0.5652	0.032*	
C11B	0.61633 (18)	0.65830 (17)	0.56501 (8)	0.0286 (4)	
H11C	0.6945	0.6147	0.5852	0.034*	
H11D	0.6112	0.6417	0.5188	0.034*	
C12B	0.63499 (19)	0.79483 (17)	0.58091 (8)	0.0305 (4)	
H12C	0.7229	0.8214	0.5690	0.037*	
H12D	0.5628	0.8389	0.5563	0.037*	
C13B	0.62968 (16)	0.82373 (16)	0.65231 (8)	0.0264 (3)	
H13C	0.7065	0.7848	0.6766	0.032*	
H13D	0.6392	0.9113	0.6613	0.032*	
C14B	0.03618 (15)	1.10034 (14)	0.92173 (7)	0.0196 (3)	
H14B	−0.0398	1.1405	0.9021	0.024*	
C15B	0.09681 (15)	1.15919 (14)	0.98310 (7)	0.0194 (3)	
C16B	0.19148 (16)	1.10511 (15)	1.02825 (7)	0.0224 (3)	
H16B	0.2201	1.0254	1.0202	0.027*	
C17B	0.24303 (16)	1.16944 (16)	1.08498 (7)	0.0259 (3)	
H17B	0.3063	1.1326	1.1144	0.031*	
C18B	0.20085 (16)	1.28832 (16)	1.09812 (8)	0.0269 (4)	
H18B	0.2365	1.3314	1.1359	0.032*	
C19B	0.10512 (17)	1.34206 (16)	1.05443 (8)	0.0257 (3)	
H19B	0.0756	1.4212	1.0632	0.031*	
C20B	0.05325 (16)	1.27844 (14)	0.99780 (8)	0.0231 (3)	
H20B	−0.0115	1.3153	0.9691	0.028*	
C21B	−0.02380 (15)	0.84445 (14)	0.72575 (7)	0.0200 (3)	
H21B	−0.0778	0.9086	0.7102	0.024*	
C22B	0.00506 (15)	0.74815 (14)	0.67780 (7)	0.0195 (3)	
C23B	−0.00327 (16)	0.62347 (15)	0.68842 (7)	0.0232 (3)	
H23B	−0.0276	0.5990	0.7275	0.028*	
C24B	0.02427 (18)	0.53589 (15)	0.64137 (8)	0.0277 (3)	
H24B	0.0165	0.4533	0.6486	0.033*	
C25B	0.06334 (18)	0.57154 (17)	0.58366 (8)	0.0302 (4)	
H25B	0.0846	0.5128	0.5527	0.036*	
C26B	0.07085 (19)	0.69446 (17)	0.57191 (8)	0.0301 (4)	
H26B	0.0966	0.7182	0.5330	0.036*	
C27B	0.03991 (16)	0.78211 (15)	0.61819 (7)	0.0246 (3)	
H27B	0.0423	0.8644	0.6095	0.029*	

### Atomic displacement parameters (Å^2^)

**Table:**

	*U*^11^	*U*^22^	*U*^33^	*U*^12^	*U*^13^	*U*^23^
O1A	0.0263 (6)	0.0261 (6)	0.0247 (5)	−0.0069 (5)	0.0054 (4)	−0.0003 (5)
O2A	0.0240 (5)	0.0310 (6)	0.0269 (6)	0.0034 (5)	0.0007 (5)	−0.0110 (5)
N1A	0.0184 (6)	0.0178 (6)	0.0180 (6)	−0.0024 (5)	0.0019 (5)	−0.0018 (5)
N2A	0.0202 (6)	0.0169 (6)	0.0197 (6)	0.0016 (5)	−0.0001 (5)	−0.0007 (5)
C1A	0.0186 (7)	0.0175 (7)	0.0204 (7)	0.0001 (6)	0.0002 (5)	0.0007 (6)
C2A	0.0179 (6)	0.0190 (7)	0.0184 (7)	0.0011 (6)	0.0006 (5)	0.0015 (6)
C3A	0.0207 (7)	0.0225 (8)	0.0178 (7)	−0.0040 (6)	0.0008 (5)	−0.0018 (6)
C4A	0.0184 (7)	0.0166 (7)	0.0212 (7)	0.0008 (6)	0.0010 (5)	0.0009 (6)
C5A	0.0168 (6)	0.0166 (7)	0.0223 (7)	0.0025 (5)	0.0023 (5)	0.0011 (6)
C6A	0.0207 (7)	0.0192 (7)	0.0187 (7)	−0.0025 (6)	0.0033 (5)	−0.0007 (6)
C7A	0.0204 (7)	0.0184 (7)	0.0191 (7)	−0.0006 (6)	0.0015 (5)	−0.0014 (6)
C8A	0.0195 (7)	0.0215 (8)	0.0204 (7)	−0.0019 (6)	0.0031 (6)	−0.0029 (6)
C9A	0.0246 (8)	0.0330 (9)	0.0274 (8)	0.0078 (7)	−0.0022 (6)	−0.0056 (7)
C10A	0.0369 (9)	0.0381 (10)	0.0261 (8)	0.0161 (8)	−0.0085 (7)	−0.0042 (7)
C11A	0.0346 (9)	0.0382 (10)	0.0263 (8)	0.0038 (8)	−0.0071 (7)	−0.0062 (8)
C12A	0.0363 (9)	0.0217 (8)	0.0240 (8)	0.0047 (7)	−0.0033 (7)	−0.0051 (6)
C13A	0.0290 (8)	0.0182 (7)	0.0220 (7)	−0.0001 (6)	−0.0018 (6)	−0.0006 (6)
C14A	0.0213 (7)	0.0169 (7)	0.0250 (7)	0.0013 (6)	0.0062 (6)	−0.0001 (6)
C15A	0.0212 (7)	0.0194 (8)	0.0217 (7)	0.0000 (6)	0.0080 (6)	−0.0009 (6)
C16A	0.0281 (8)	0.0189 (7)	0.0230 (7)	0.0025 (6)	0.0078 (6)	0.0021 (6)
C17A	0.0323 (8)	0.0315 (9)	0.0196 (7)	0.0004 (7)	0.0060 (6)	0.0000 (7)
C18A	0.0381 (9)	0.0285 (9)	0.0246 (8)	−0.0037 (8)	0.0065 (7)	−0.0079 (7)
C19A	0.0402 (9)	0.0175 (8)	0.0322 (9)	0.0012 (7)	0.0079 (7)	−0.0031 (7)
C20A	0.0306 (8)	0.0193 (8)	0.0249 (8)	0.0049 (6)	0.0044 (6)	0.0006 (6)
C21A	0.0194 (7)	0.0195 (7)	0.0199 (7)	−0.0001 (6)	0.0012 (5)	0.0013 (6)
C22A	0.0165 (6)	0.0201 (7)	0.0198 (7)	0.0012 (6)	−0.0033 (5)	−0.0010 (6)
C23A	0.0222 (7)	0.0218 (8)	0.0246 (7)	−0.0008 (6)	−0.0037 (6)	−0.0003 (7)
C24A	0.0266 (8)	0.0219 (8)	0.0272 (8)	0.0025 (6)	−0.0074 (6)	−0.0058 (6)
C25A	0.0237 (7)	0.0319 (9)	0.0229 (7)	0.0072 (7)	−0.0031 (6)	−0.0086 (7)
C26A	0.0219 (7)	0.0330 (9)	0.0208 (7)	0.0012 (7)	−0.0004 (6)	−0.0015 (7)
C27A	0.0201 (7)	0.0230 (8)	0.0227 (7)	−0.0001 (6)	−0.0011 (6)	0.0004 (6)
O1B	0.0258 (6)	0.0239 (6)	0.0252 (5)	0.0068 (5)	−0.0008 (4)	0.0005 (5)
O2B	0.0237 (6)	0.0344 (7)	0.0288 (6)	−0.0084 (5)	0.0044 (5)	−0.0121 (5)
N1B	0.0190 (6)	0.0191 (6)	0.0173 (6)	0.0010 (5)	0.0018 (5)	−0.0026 (5)
N2B	0.0195 (6)	0.0167 (6)	0.0190 (6)	−0.0019 (5)	0.0031 (5)	−0.0012 (5)
C1B	0.0184 (7)	0.0180 (7)	0.0206 (7)	−0.0001 (6)	0.0027 (5)	0.0022 (6)
C2B	0.0172 (6)	0.0164 (7)	0.0215 (7)	−0.0027 (5)	0.0028 (5)	0.0015 (6)
C3B	0.0197 (7)	0.0162 (7)	0.0211 (7)	−0.0009 (6)	0.0028 (6)	0.0009 (6)
C4B	0.0213 (7)	0.0208 (7)	0.0170 (7)	0.0020 (6)	0.0030 (5)	−0.0025 (6)
C5B	0.0184 (6)	0.0182 (7)	0.0182 (7)	−0.0009 (6)	0.0032 (5)	0.0019 (5)
C6B	0.0199 (7)	0.0191 (7)	0.0201 (7)	0.0010 (6)	0.0009 (6)	−0.0010 (6)
C7B	0.0206 (7)	0.0185 (7)	0.0197 (7)	−0.0005 (6)	0.0024 (5)	−0.0024 (6)
C8B	0.0198 (7)	0.0216 (8)	0.0204 (7)	−0.0007 (6)	0.0026 (6)	−0.0028 (6)
C9B	0.0275 (8)	0.0173 (7)	0.0226 (7)	0.0000 (6)	0.0054 (6)	−0.0008 (6)
C10B	0.0328 (8)	0.0223 (8)	0.0239 (8)	−0.0057 (7)	0.0042 (7)	−0.0042 (6)
C11B	0.0303 (8)	0.0331 (9)	0.0226 (8)	−0.0019 (7)	0.0076 (6)	−0.0055 (7)
C12B	0.0317 (8)	0.0328 (9)	0.0288 (8)	−0.0076 (7)	0.0122 (7)	−0.0002 (7)
C13B	0.0240 (8)	0.0273 (9)	0.0282 (8)	−0.0079 (7)	0.0073 (6)	−0.0049 (7)
C14B	0.0194 (7)	0.0196 (7)	0.0203 (7)	0.0003 (6)	0.0034 (6)	0.0018 (6)
C15B	0.0190 (7)	0.0205 (7)	0.0196 (7)	−0.0036 (6)	0.0072 (6)	−0.0005 (6)
C16B	0.0228 (7)	0.0233 (8)	0.0215 (7)	−0.0007 (6)	0.0059 (6)	−0.0015 (6)
C17B	0.0222 (7)	0.0343 (9)	0.0210 (7)	−0.0023 (7)	0.0034 (6)	−0.0015 (7)
C18B	0.0261 (8)	0.0320 (9)	0.0231 (7)	−0.0103 (7)	0.0096 (6)	−0.0071 (7)
C19B	0.0289 (8)	0.0217 (8)	0.0276 (8)	−0.0042 (7)	0.0124 (7)	−0.0066 (6)
C20B	0.0246 (7)	0.0218 (8)	0.0236 (7)	0.0006 (6)	0.0069 (6)	−0.0003 (7)
C21B	0.0182 (7)	0.0164 (7)	0.0246 (7)	−0.0005 (6)	−0.0009 (6)	0.0015 (6)
C22B	0.0184 (7)	0.0181 (7)	0.0213 (7)	−0.0024 (6)	−0.0009 (5)	−0.0006 (6)
C23B	0.0272 (8)	0.0203 (8)	0.0218 (7)	−0.0048 (6)	0.0021 (6)	0.0001 (6)
C24B	0.0337 (9)	0.0187 (8)	0.0293 (8)	−0.0015 (7)	−0.0010 (7)	−0.0011 (6)
C25B	0.0362 (9)	0.0287 (9)	0.0241 (8)	0.0055 (7)	−0.0001 (7)	−0.0047 (7)
C26B	0.0362 (9)	0.0340 (10)	0.0206 (8)	0.0044 (8)	0.0044 (7)	0.0042 (7)
C27B	0.0284 (8)	0.0213 (8)	0.0233 (7)	−0.0002 (6)	−0.0014 (6)	0.0044 (6)

### Geometric parameters (Å, °)

**Table:**

O1A—C1A	1.2240 (18)	O1B—C1B	1.2231 (18)
O2A—C6A	1.2287 (18)	O2B—C6B	1.2313 (18)
N1A—C6A	1.3597 (19)	N1B—C6B	1.3619 (19)
N1A—C3A	1.4587 (18)	N1B—C4B	1.4605 (18)
N1A—C4A	1.4651 (18)	N1B—C3B	1.4606 (18)
N2A—C9A	1.458 (2)	N2B—C13B	1.4647 (19)
N2A—C13A	1.462 (2)	N2B—C9B	1.466 (2)
N2A—C8A	1.4679 (19)	N2B—C8B	1.4685 (19)
C1A—C5A	1.498 (2)	C1B—C2B	1.497 (2)
C1A—C2A	1.503 (2)	C1B—C5B	1.501 (2)
C2A—C21A	1.349 (2)	C2B—C21B	1.344 (2)
C2A—C3A	1.511 (2)	C2B—C3B	1.506 (2)
C3A—H3AA	0.9700	C3B—H3BA	0.9700
C3A—H3AB	0.9700	C3B—H3BB	0.9700
C4A—C5A	1.506 (2)	C4B—C5B	1.516 (2)
C4A—H4AA	0.9700	C4B—H4BA	0.9700
C4A—H4AB	0.9700	C4B—H4BB	0.9700
C5A—C14A	1.345 (2)	C5B—C14B	1.348 (2)
C6A—C7A	1.520 (2)	C6B—C7B	1.517 (2)
C7A—C8A	1.519 (2)	C7B—C8B	1.522 (2)
C7A—H7AA	0.9700	C7B—H7BA	0.9700
C7A—H7AB	0.9700	C7B—H7BB	0.9700
C8A—H8AA	0.9700	C8B—H8BA	0.9700
C8A—H8AB	0.9700	C8B—H8BB	0.9700
C9A—C10A	1.516 (2)	C9B—C10B	1.526 (2)
C9A—H9AA	0.9700	C9B—H9BA	0.9700
C9A—H9AB	0.9700	C9B—H9BB	0.9700
C10A—C11A	1.521 (3)	C10B—C11B	1.523 (2)
C10A—H10A	0.9700	C10B—H10C	0.9700
C10A—H10B	0.9700	C10B—H10D	0.9700
C11A—C12A	1.518 (2)	C11B—C12B	1.521 (3)
C11A—H11A	0.9700	C11B—H11C	0.9700
C11A—H11B	0.9700	C11B—H11D	0.9700
C12A—C13A	1.522 (2)	C12B—C13B	1.518 (2)
C12A—H12A	0.9700	C12B—H12C	0.9700
C12A—H12B	0.9700	C12B—H12D	0.9700
C13A—H13A	0.9700	C13B—H13C	0.9700
C13A—H13B	0.9700	C13B—H13D	0.9700
C14A—C15A	1.468 (2)	C14B—C15B	1.467 (2)
C14A—H14A	0.9300	C14B—H14B	0.9300
C15A—C16A	1.398 (2)	C15B—C16B	1.401 (2)
C15A—C20A	1.402 (2)	C15B—C20B	1.404 (2)
C16A—C17A	1.384 (2)	C16B—C17B	1.390 (2)
C16A—H16A	0.9300	C16B—H16B	0.9300
C17A—C18A	1.389 (3)	C17B—C18B	1.390 (2)
C17A—H17A	0.9300	C17B—H17B	0.9300
C18A—C19A	1.385 (2)	C18B—C19B	1.387 (2)
C18A—H18A	0.9300	C18B—H18B	0.9300
C19A—C20A	1.391 (2)	C19B—C20B	1.386 (2)
C19A—H19A	0.9300	C19B—H19B	0.9300
C20A—H20A	0.9300	C20B—H20B	0.9300
C21A—C22A	1.464 (2)	C21B—C22B	1.467 (2)
C21A—H21A	0.9300	C21B—H21B	0.9300
C22A—C27A	1.401 (2)	C22B—C23B	1.399 (2)
C22A—C23A	1.408 (2)	C22B—C27B	1.400 (2)
C23A—C24A	1.386 (2)	C23B—C24B	1.387 (2)
C23A—H23A	0.9300	C23B—H23B	0.9300
C24A—C25A	1.384 (2)	C24B—C25B	1.384 (2)
C24A—H24A	0.9300	C24B—H24B	0.9300
C25A—C26A	1.388 (2)	C25B—C26B	1.385 (3)
C25A—H25A	0.9300	C25B—H25B	0.9300
C26A—C27A	1.391 (2)	C26B—C27B	1.386 (2)
C26A—H26A	0.9300	C26B—H26B	0.9300
C27A—H27A	0.9300	C27B—H27B	0.9300
			
C6A—N1A—C3A	120.39 (12)	C6B—N1B—C4B	120.11 (12)
C6A—N1A—C4A	123.51 (12)	C6B—N1B—C3B	123.27 (12)
C3A—N1A—C4A	112.84 (12)	C4B—N1B—C3B	113.00 (12)
C9A—N2A—C13A	110.03 (13)	C13B—N2B—C9B	110.16 (12)
C9A—N2A—C8A	109.42 (12)	C13B—N2B—C8B	108.53 (12)
C13A—N2A—C8A	112.11 (12)	C9B—N2B—C8B	111.32 (12)
O1A—C1A—C5A	120.53 (13)	O1B—C1B—C2B	120.59 (13)
O1A—C1A—C2A	121.82 (13)	O1B—C1B—C5B	121.54 (13)
C5A—C1A—C2A	117.63 (13)	C2B—C1B—C5B	117.85 (13)
C21A—C2A—C1A	115.99 (13)	C21B—C2B—C1B	117.64 (14)
C21A—C2A—C3A	124.80 (13)	C21B—C2B—C3B	125.72 (14)
C1A—C2A—C3A	119.21 (13)	C1B—C2B—C3B	116.01 (12)
N1A—C3A—C2A	112.08 (12)	N1B—C3B—C2B	106.82 (12)
N1A—C3A—H3AA	109.2	N1B—C3B—H3BA	110.4
C2A—C3A—H3AA	109.2	C2B—C3B—H3BA	110.4
N1A—C3A—H3AB	109.2	N1B—C3B—H3BB	110.4
C2A—C3A—H3AB	109.2	C2B—C3B—H3BB	110.4
H3AA—C3A—H3AB	107.9	H3BA—C3B—H3BB	108.6
N1A—C4A—C5A	107.02 (12)	N1B—C4B—C5B	111.91 (12)
N1A—C4A—H4AA	110.3	N1B—C4B—H4BA	109.2
C5A—C4A—H4AA	110.3	C5B—C4B—H4BA	109.2
N1A—C4A—H4AB	110.3	N1B—C4B—H4BB	109.2
C5A—C4A—H4AB	110.3	C5B—C4B—H4BB	109.2
H4AA—C4A—H4AB	108.6	H4BA—C4B—H4BB	107.9
C14A—C5A—C1A	117.45 (14)	C14B—C5B—C1B	116.31 (13)
C14A—C5A—C4A	125.86 (14)	C14B—C5B—C4B	124.71 (13)
C1A—C5A—C4A	116.06 (12)	C1B—C5B—C4B	118.98 (12)
O2A—C6A—N1A	121.69 (13)	O2B—C6B—N1B	121.60 (13)
O2A—C6A—C7A	121.87 (13)	O2B—C6B—C7B	121.71 (14)
N1A—C6A—C7A	116.43 (13)	N1B—C6B—C7B	116.68 (13)
C8A—C7A—C6A	112.45 (12)	C6B—C7B—C8B	112.32 (12)
C8A—C7A—H7AA	109.1	C6B—C7B—H7BA	109.1
C6A—C7A—H7AA	109.1	C8B—C7B—H7BA	109.1
C8A—C7A—H7AB	109.1	C6B—C7B—H7BB	109.1
C6A—C7A—H7AB	109.1	C8B—C7B—H7BB	109.1
H7AA—C7A—H7AB	107.8	H7BA—C7B—H7BB	107.9
N2A—C8A—C7A	111.46 (12)	N2B—C8B—C7B	111.72 (12)
N2A—C8A—H8AA	109.3	N2B—C8B—H8BA	109.3
C7A—C8A—H8AA	109.3	C7B—C8B—H8BA	109.3
N2A—C8A—H8AB	109.3	N2B—C8B—H8BB	109.3
C7A—C8A—H8AB	109.3	C7B—C8B—H8BB	109.3
H8AA—C8A—H8AB	108.0	H8BA—C8B—H8BB	107.9
N2A—C9A—C10A	111.35 (13)	N2B—C9B—C10B	110.92 (13)
N2A—C9A—H9AA	109.4	N2B—C9B—H9BA	109.5
C10A—C9A—H9AA	109.4	C10B—C9B—H9BA	109.5
N2A—C9A—H9AB	109.4	N2B—C9B—H9BB	109.5
C10A—C9A—H9AB	109.4	C10B—C9B—H9BB	109.5
H9AA—C9A—H9AB	108.0	H9BA—C9B—H9BB	108.0
C9A—C10A—C11A	110.56 (15)	C11B—C10B—C9B	110.95 (13)
C9A—C10A—H10A	109.5	C11B—C10B—H10C	109.4
C11A—C10A—H10A	109.5	C9B—C10B—H10C	109.4
C9A—C10A—H10B	109.5	C11B—C10B—H10D	109.4
C11A—C10A—H10B	109.5	C9B—C10B—H10D	109.4
H10A—C10A—H10B	108.1	H10C—C10B—H10D	108.0
C12A—C11A—C10A	109.57 (14)	C12B—C11B—C10B	109.34 (14)
C12A—C11A—H11A	109.8	C12B—C11B—H11C	109.8
C10A—C11A—H11A	109.8	C10B—C11B—H11C	109.8
C12A—C11A—H11B	109.8	C12B—C11B—H11D	109.8
C10A—C11A—H11B	109.8	C10B—C11B—H11D	109.8
H11A—C11A—H11B	108.2	H11C—C11B—H11D	108.3
C11A—C12A—C13A	111.06 (14)	C13B—C12B—C11B	110.47 (14)
C11A—C12A—H12A	109.4	C13B—C12B—H12C	109.6
C13A—C12A—H12A	109.4	C11B—C12B—H12C	109.6
C11A—C12A—H12B	109.4	C13B—C12B—H12D	109.6
C13A—C12A—H12B	109.4	C11B—C12B—H12D	109.6
H12A—C12A—H12B	108.0	H12C—C12B—H12D	108.1
N2A—C13A—C12A	111.09 (13)	N2B—C13B—C12B	112.04 (13)
N2A—C13A—H13A	109.4	N2B—C13B—H13C	109.2
C12A—C13A—H13A	109.4	C12B—C13B—H13C	109.2
N2A—C13A—H13B	109.4	N2B—C13B—H13D	109.2
C12A—C13A—H13B	109.4	C12B—C13B—H13D	109.2
H13A—C13A—H13B	108.0	H13C—C13B—H13D	107.9
C5A—C14A—C15A	128.01 (15)	C5B—C14B—C15B	131.29 (14)
C5A—C14A—H14A	116.0	C5B—C14B—H14B	114.4
C15A—C14A—H14A	116.0	C15B—C14B—H14B	114.4
C16A—C15A—C20A	118.67 (14)	C16B—C15B—C20B	117.97 (14)
C16A—C15A—C14A	118.11 (14)	C16B—C15B—C14B	125.31 (14)
C20A—C15A—C14A	123.21 (14)	C20B—C15B—C14B	116.70 (14)
C17A—C16A—C15A	121.01 (15)	C17B—C16B—C15B	120.63 (15)
C17A—C16A—H16A	119.5	C17B—C16B—H16B	119.7
C15A—C16A—H16A	119.5	C15B—C16B—H16B	119.7
C16A—C17A—C18A	119.62 (16)	C18B—C17B—C16B	120.60 (16)
C16A—C17A—H17A	120.2	C18B—C17B—H17B	119.7
C18A—C17A—H17A	120.2	C16B—C17B—H17B	119.7
C19A—C18A—C17A	120.32 (15)	C19B—C18B—C17B	119.36 (15)
C19A—C18A—H18A	119.8	C19B—C18B—H18B	120.3
C17A—C18A—H18A	119.8	C17B—C18B—H18B	120.3
C18A—C19A—C20A	120.13 (16)	C20B—C19B—C18B	120.34 (15)
C18A—C19A—H19A	119.9	C20B—C19B—H19B	119.8
C20A—C19A—H19A	119.9	C18B—C19B—H19B	119.8
C19A—C20A—C15A	120.18 (15)	C19B—C20B—C15B	121.08 (15)
C19A—C20A—H20A	119.9	C19B—C20B—H20B	119.5
C15A—C20A—H20A	119.9	C15B—C20B—H20B	119.5
C2A—C21A—C22A	131.26 (14)	C2B—C21B—C22B	128.10 (14)
C2A—C21A—H21A	114.4	C2B—C21B—H21B	116.0
C22A—C21A—H21A	114.4	C22B—C21B—H21B	116.0
C27A—C22A—C23A	117.67 (14)	C23B—C22B—C27B	118.29 (14)
C27A—C22A—C21A	125.49 (14)	C23B—C22B—C21B	123.00 (14)
C23A—C22A—C21A	116.82 (14)	C27B—C22B—C21B	118.70 (14)
C24A—C23A—C22A	121.14 (15)	C24B—C23B—C22B	120.79 (15)
C24A—C23A—H23A	119.4	C24B—C23B—H23B	119.6
C22A—C23A—H23A	119.4	C22B—C23B—H23B	119.6
C25A—C24A—C23A	120.03 (15)	C25B—C24B—C23B	119.95 (16)
C25A—C24A—H24A	120.0	C25B—C24B—H24B	120.0
C23A—C24A—H24A	120.0	C23B—C24B—H24B	120.0
C24A—C25A—C26A	120.10 (15)	C24B—C25B—C26B	120.17 (15)
C24A—C25A—H25A	120.0	C24B—C25B—H25B	119.9
C26A—C25A—H25A	120.0	C26B—C25B—H25B	119.9
C25A—C26A—C27A	119.93 (16)	C25B—C26B—C27B	119.97 (16)
C25A—C26A—H26A	120.0	C25B—C26B—H26B	120.0
C27A—C26A—H26A	120.0	C27B—C26B—H26B	120.0
C26A—C27A—C22A	121.10 (15)	C26B—C27B—C22B	120.76 (15)
C26A—C27A—H27A	119.5	C26B—C27B—H27B	119.6
C22A—C27A—H27A	119.5	C22B—C27B—H27B	119.6
			
O1A—C1A—C2A—C21A	−1.8 (2)	O1B—C1B—C2B—C21B	23.1 (2)
C5A—C1A—C2A—C21A	−179.72 (13)	C5B—C1B—C2B—C21B	−155.47 (14)
O1A—C1A—C2A—C3A	178.36 (14)	O1B—C1B—C2B—C3B	−165.49 (13)
C5A—C1A—C2A—C3A	0.40 (19)	C5B—C1B—C2B—C3B	15.92 (19)
C6A—N1A—C3A—C2A	−106.24 (15)	C6B—N1B—C3B—C2B	−89.04 (16)
C4A—N1A—C3A—C2A	54.07 (17)	C4B—N1B—C3B—C2B	69.48 (15)
C21A—C2A—C3A—N1A	161.89 (14)	C21B—C2B—C3B—N1B	122.30 (16)
C1A—C2A—C3A—N1A	−18.23 (19)	C1B—C2B—C3B—N1B	−48.30 (16)
C6A—N1A—C4A—C5A	90.49 (16)	C6B—N1B—C4B—C5B	105.26 (15)
C3A—N1A—C4A—C5A	−69.11 (15)	C3B—N1B—C4B—C5B	−54.02 (17)
O1A—C1A—C5A—C14A	−22.7 (2)	O1B—C1B—C5B—C14B	1.2 (2)
C2A—C1A—C5A—C14A	155.34 (14)	C2B—C1B—C5B—C14B	179.79 (13)
O1A—C1A—C5A—C4A	165.93 (14)	O1B—C1B—C5B—C4B	−178.41 (14)
C2A—C1A—C5A—C4A	−16.08 (19)	C2B—C1B—C5B—C4B	0.16 (19)
N1A—C4A—C5A—C14A	−122.52 (16)	N1B—C4B—C5B—C14B	−162.00 (14)
N1A—C4A—C5A—C1A	48.07 (16)	N1B—C4B—C5B—C1B	17.59 (19)
C3A—N1A—C6A—O2A	−9.6 (2)	C4B—N1B—C6B—O2B	10.5 (2)
C4A—N1A—C6A—O2A	−167.72 (14)	C3B—N1B—C6B—O2B	167.53 (14)
C3A—N1A—C6A—C7A	171.02 (13)	C4B—N1B—C6B—C7B	−170.41 (13)
C4A—N1A—C6A—C7A	12.9 (2)	C3B—N1B—C6B—C7B	−13.3 (2)
O2A—C6A—C7A—C8A	−4.9 (2)	O2B—C6B—C7B—C8B	5.0 (2)
N1A—C6A—C7A—C8A	174.48 (13)	N1B—C6B—C7B—C8B	−174.17 (13)
C9A—N2A—C8A—C7A	−172.16 (14)	C13B—N2B—C8B—C7B	172.96 (13)
C13A—N2A—C8A—C7A	65.48 (17)	C9B—N2B—C8B—C7B	−65.63 (16)
C6A—C7A—C8A—N2A	157.16 (13)	C6B—C7B—C8B—N2B	−158.65 (12)
C13A—N2A—C9A—C10A	−60.41 (19)	C13B—N2B—C9B—C10B	−58.88 (17)
C8A—N2A—C9A—C10A	175.99 (15)	C8B—N2B—C9B—C10B	−179.33 (12)
N2A—C9A—C10A—C11A	58.0 (2)	N2B—C9B—C10B—C11B	57.66 (18)
C9A—C10A—C11A—C12A	−53.8 (2)	C9B—C10B—C11B—C12B	−54.71 (19)
C10A—C11A—C12A—C13A	53.5 (2)	C10B—C11B—C12B—C13B	54.11 (19)
C9A—N2A—C13A—C12A	59.63 (17)	C9B—N2B—C13B—C12B	59.22 (18)
C8A—N2A—C13A—C12A	−178.35 (13)	C8B—N2B—C13B—C12B	−178.67 (14)
C11A—C12A—C13A—N2A	−56.95 (19)	C11B—C12B—C13B—N2B	−57.29 (19)
C1A—C5A—C14A—C15A	−174.96 (14)	C1B—C5B—C14B—C15B	178.74 (14)
C4A—C5A—C14A—C15A	−4.5 (3)	C4B—C5B—C14B—C15B	−1.7 (3)
C5A—C14A—C15A—C16A	135.26 (17)	C5B—C14B—C15B—C16B	−14.2 (3)
C5A—C14A—C15A—C20A	−46.2 (2)	C5B—C14B—C15B—C20B	167.28 (16)
C20A—C15A—C16A—C17A	2.7 (2)	C20B—C15B—C16B—C17B	−1.7 (2)
C14A—C15A—C16A—C17A	−178.70 (14)	C14B—C15B—C16B—C17B	179.77 (14)
C15A—C16A—C17A—C18A	−2.6 (2)	C15B—C16B—C17B—C18B	0.4 (2)
C16A—C17A—C18A—C19A	0.4 (3)	C16B—C17B—C18B—C19B	0.8 (2)
C17A—C18A—C19A—C20A	1.7 (3)	C17B—C18B—C19B—C20B	−0.8 (2)
C18A—C19A—C20A—C15A	−1.6 (3)	C18B—C19B—C20B—C15B	−0.5 (2)
C16A—C15A—C20A—C19A	−0.6 (2)	C16B—C15B—C20B—C19B	1.8 (2)
C14A—C15A—C20A—C19A	−179.14 (15)	C14B—C15B—C20B—C19B	−179.60 (14)
C1A—C2A—C21A—C22A	−178.37 (15)	C1B—C2B—C21B—C22B	176.08 (14)
C3A—C2A—C21A—C22A	1.5 (3)	C3B—C2B—C21B—C22B	5.6 (3)
C2A—C21A—C22A—C27A	15.4 (3)	C2B—C21B—C22B—C23B	44.3 (2)
C2A—C21A—C22A—C23A	−166.71 (16)	C2B—C21B—C22B—C27B	−137.01 (17)
C27A—C22A—C23A—C24A	−2.0 (2)	C27B—C22B—C23B—C24B	0.9 (2)
C21A—C22A—C23A—C24A	179.93 (14)	C21B—C22B—C23B—C24B	179.62 (15)
C22A—C23A—C24A—C25A	0.6 (2)	C22B—C23B—C24B—C25B	1.4 (2)
C23A—C24A—C25A—C26A	0.8 (2)	C23B—C24B—C25B—C26B	−2.0 (3)
C24A—C25A—C26A—C27A	−0.7 (2)	C24B—C25B—C26B—C27B	0.4 (3)
C25A—C26A—C27A—C22A	−0.8 (2)	C25B—C26B—C27B—C22B	2.0 (3)
C23A—C22A—C27A—C26A	2.1 (2)	C23B—C22B—C27B—C26B	−2.6 (2)
C21A—C22A—C27A—C26A	179.97 (14)	C21B—C22B—C27B—C26B	178.64 (15)

### Hydrogen-bond geometry (Å, °)

**Table:**

*D*—H···*A*	*D*—H	H···*A*	*D*···*A*	*D*—H···*A*
C7A—H7AB···O1B^i^	0.97	2.59	3.3737 (19)	138
C19B—H19B···O2A^ii^	0.93	2.44	3.233 (2)	143
C24A—H24A···O2B^iii^	0.93	2.40	3.190 (2)	143
C24B—H24B···N2A	0.93	2.61	3.411 (2)	145

Symmetry codes: (i) *x*, *y*−1, *z*; (ii) −*x*, −*y*+2, −*z*+2; (iii) −*x*+1, −*y*+2, −*z*+2.
